# Targeted Imaging of Endometriosis and Image-Guided Resection of Lesions Using Gonadotropin-Releasing Hormone Analogue-Modified Indocyanine Green

**DOI:** 10.1155/2023/6674054

**Published:** 2023-12-04

**Authors:** Jing Peng, Qiyu Liu, Tao Pu, Mingxing Zhang, Meng Zhang, Ming Du, Guiling Li, Xiaoyan Zhang, Congjian Xu

**Affiliations:** ^1^Obstetrics and Gynecology Hospital, Fudan University, Shanghai 200011, China; ^2^Shanghai Key Laboratory of Female Reproductive Endocrine Related Diseases, Shanghai 200011, China; ^3^Department of Obstetrics and Gynecology of Shanghai Medical School, Fudan University, Shanghai 200032, China

## Abstract

**Objective:**

In this study, we utilized gonadotropin-releasing hormone analogue-modified indocyanine green (GnRHa-ICG) to improve the accuracy of intraoperative recognition and resection of endometriotic lesions.

**Methods:**

Gonadotropin-releasing hormone receptor (GnRHR) expression was detected in endometriosis tissues and cell lines via immunohistochemistry and western blotting. The in vitro binding capacities of GnRHa, GnRHa-ICG, and ICG were determined using fluorescence microscopy and flow cytometry. In vivo imaging was performed in mouse models of endometriosis using a near-infrared fluorescence (NIRF) imaging system and fluorescence navigation system. The ex vivo binding capacity was determined using confocal fluorescence microscopy.

**Results:**

GnRHa-ICG exhibited a significantly stronger binding capacity to endometriotic cells and tissues than ICG. In mice with endometriosis, GnRHa-ICG specifically imaged endometriotic tissues (EMTs) after intraperitoneal administration, whereas ICG exhibited signals in the intestine. GnRHa-ICG showed the highest fluorescence signals in the EMTs at 2 h and a good signal-to-noise ratio at 48 h postadministration. Compared with traditional surgery under white light, targeted NIRF imaging-guided surgery completely resected endometriotic lesions with a sensitivity of 97.3% and specificity of 77.8%. No obvious toxicity was observed in routine blood tests, serum biochemicals, or histopathology in mice.

**Conclusions:**

GnRHa-ICG specifically recognized and localized endometriotic lesions and guided complete resection of lesions with high accuracy.

## 1. Introduction

Endometriosis is a common benign gynecological disease that seriously affects the physical and mental health of women of childbearing age. Laparoscopic surgery is currently the dominant approach for relieving symptoms and promoting fertility in patients with endometriosis [1]. However, the recurrence rate is as high as 40-50% within 5 years of surgery [2]. Recurrent endometriotic lesions can arise from incompletely removed or unrecognized lesions [2–4]. To effectively reduce the recurrence rate, accurate intraoperative identification and complete resection of endometriotic lesions are critical [5–7].

Traditional imaging approaches such as ultrasound [8–10] and MRI [11, 12] lack sufficient sensitivity and specificity for endometriosis, and it is difficult to recognize subtle lesions and provide real-time intraoperative imaging [8]. As an emerging imaging technology, NIRF imaging has been widely employed in clinical practice in recent years and is more suitable for intraoperative real-time imaging because of its deeper tissue penetration and weaker autofluorescence background signals [13].

Indocyanine green (ICG), the only clinically approved near-infrared (NIR) fluorophore, has been used in intraoperative imaging for endometriosis, but it cannot improve the detection of endometriotic lesions compared to traditional white light imaging owing to its nonspecificity [14]. Specific imaging agents are required to detect endometriosis.

GnRHR is mainly expressed in the pituitary and pelvic-abdominal reproductive systems [15], and a high expression of GnRHR has been reported in endometriotic cells and tissues [16–19]. Hence, GnRHR is a promising imaging target in endometriosis. As the binding ligands of GnRHR, GnRH analogues (both agonists and antagonists) have been proven to have a high affinity for tumor cells [20]. Conjugates of GnRH analogues with cytotoxic drugs have shown potential for targeted cancer therapy [21]. The ^111^In-labeled GnRH peptides effectively detected human prostate cancer [22] and breast cancer [23]. Our previous study indicated that GnRHa-modified ICG can specifically recognize and image peritoneal metastases in ovarian cancer [24].

In this study, the clinical value of GnRHa-ICG was first investigated in endometriosis. The expression of GnRHR was detected in endometriosis tissues and cell lines to evaluate the feasibility of GnRHR as an imaging target for endometriosis, and subsequently, the binding capacities of GnRHa, GnRHa-ICG, and ICG were determined in vitro and in vivo. The performance of intraoperative imaging and image-guided resection was assessed in mouse models of endometriosis. The biodistribution and safety of GnRHa-ICG were investigated in vivo.

## 2. Materials and Methods

### 2.1. Cell Culture

The immortalized human endometriotic epithelial cell line 12Z (cat. No. T0764) and human immortalized endometrial stromal cells (ESC) (cat. No T0533) purchased from ABM (Richmond, BC, Canada) were used for the experiments. The 12Z cells were cultured in Prigrow III medium (Cat. No TM003, ABM), and HESC were cultured in DMEM/F-12 medium, both of which were supplemented with 10% FBS, 100 U/mL penicillin, and 100 *μ*g/mL streptomycin. Cells were grown at 37°C, 5% CO2 in a humidified environment.

### 2.2. Immunohistochemistry

Human tissue samples comprising 45 ovarian endometriomas, 17 deep infiltrating endometrioses, and 6 normal endometria samples were obtained from the human tissue bank of the Obstetrics and Gynecology Hospital of Fudan University with the Ethics Committee's approval. Formalin-fixed and paraffin-embedded sections were stained with hematoxylin-eosin (HE). For immunohistochemistry (IHC), samples were incubated with a 1 : 100 dilution of GnRHR antibody (ab183079, Abcam) and then incubated with a 1 : 500 dilution of secondary antibody (anti-rabbit HRP IgG, 111-035-003, Jackson ImmunoResearch). DAB substrate solution (8059, CST) was used as the chromogen.

### 2.3. Western Blot

Western blot analysis was performed as described [24]. Proteins were visualized by chemiluminescence using the ImageQuant LAS4000 system (GE Healthcare), and each immunoblot was performed at least three times.

### 2.4. Fluorescence Microscopy

The cells were cultured to 60–70% confluence in an 8-well chamber slide (Ibidi). GnRHa peptide was synthesized as the sequence of GnRH antagonist cetrorelix, and the N-terminus was labeled with fluorescein isothiocyanate (FITC) to synthesize GnRHa-FITC (GL Biochem Ltd., Shanghai, China). 12Z cells were incubated with 10 *μ*mol/L GnRHa-FITC for 60 min at 37°C, and 12Z and HESC cells were incubated with 10 *μ*mol/L GnRHa-ICG or ICG (Sigma-Aldrich) for 30 min at 37°C. After incubation, the cells were washed 3 times with PBS and fixed in 4% paraformaldehyde for 10 min, mounted with DAPI. TCS SP5 confocal microscope (Leica) was used to analyze the samples (excitation wavelength 633 nm and emission 780 nm long-pass filter, ×100 oil objective, ×1000 magnification), and ImageJ software (version 1.50 g) was used to calculate the mean fluorescence intensity for quantitative analysis.

### 2.5. Flow Cytometry

12Z and HESC cells were cultured to 70-80% confluency in 6-well plates. 12Z cells were first incubated with 10 *μ*mol/L GnRHa-FITC for 60 min at 37°C, and 12Z and HESC cells were incubated with GnRHa-ICG/ICG (10 *μ*mol/L) for 30 min at 37°C. For the blocking experiments, 12Z cells were pretreated with 100 *μ*mol/L GnRHa peptide for 10 min and then incubated with 10 *μ*mol/L GnRHa-ICG for 30 min at 37°C. The samples were measured on a CytoFLEX flow cytometer (Beckman Coulter). All the samples were examined in triplicate. The data were analyzed using FlowJo software (version X 10.0.7).

### 2.6. Cell Viability Assay

12Z cells were cultured to 40–50% confluence in 96-well plates. The cells incubated with GnRHa-ICG were divided into four groups at different concentrations (0, 1, 10, and 100 *μ*mol/L), and each group contained four subwells. Cell viability was assessed after exposure for 48 h using the Cell Counting Kit-8 assay (Dojindo). Absorbance at 450 nm (reference wavelength: 630 nm) was measured using a microplate reader.

### 2.7. Animal Model

Six- to eight-week-old female green fluorescent protein- (GFP-) transgenic C57BL/6 mice (Slack Experimental Animals Co. LT; Shanghai, China) were used in this study. All mice were maintained under controlled conditions with a light/dark (12/12 h) cycle and had ad libitum access to chow and water. The animal experiments were approved by the Institutional Animal Care and Use Committee of Fudan University. GFP transgenic C57BL/6 mice were selected as donors of uterine tissue fragments and were initially treated i.m. with 100 mg/kg estradiol benzoate (Animal Medicine Factory, Hangzhou, China) twice a week after one week of acclimation. Wild-type female C57BL/6 mice were designated as recipients. One week after treatment with estrogen, the uteri from the sacrificed donor mice were harvested in a petri dish, washed twice with sterile saline, and then split longitudinally. Finally, the uterine tissues were minced into fragments with diameters smaller than 1 mm, and then, the uterine fragments were suspended in sterile saline and injected into the abdominal cavities of recipient mice. The uterus of each donor mouse was injected in equal amounts into two recipient mice. The successful establishment of the model was indicated when nodules were palpable from the abdominal wall after 3–4 weeks of induction.

### 2.8. Near-Infrared Fluorescence Imaging

The synthesis and characterization of GnRHa-ICG are described in the study by Liu et al. [24]. The endometriotic model mice were sacrificed at the indicated times after intraperitoneal injection of GnRHa-ICG (1 mg/kg) or ICG (0.37 mg/kg), and the GFP and NIRF signals were measured using the IVIS Lumina K imaging system (PerkinElmer, Waltham, US). Each group had 3 mice, and the probe was administered after 24 h of fasting. NIRF images were obtained using a 780 nm excitation filter and an 845 nm emission filter. For ex vivo imaging, allografts and organs were dissected and analyzed immediately after sacrifice. Fluorescence signals were quantified as the average radiant efficiency ([p/s/cm^2^/sr]/[*μ*W/cm^2^]) using Living Image software. Fluorescence intensity was measured by drawing a region of interest (ROI) around the area. The signal-to-noise ratio (SNR) was calculated as the average fluorescence intensity of the tumor divided by that of skeletal muscle or intestine.

### 2.9. Target Image-Guided Resection of Endometriotic Foci

The targeted probe GnRHa-ICG (1 mg/kg) was injected intraperitoneally into model mice at the indicated times, and the abdominal cavities were exposed after sacrifice. NIRF image- and white light-guided surgeries were performed using a clinically used fluorescence navigation system (FloNavi, Optomedic Technique Inc., Guangdong, China). Endometriotic foci were resected under white light and NIRF guidance (*n* = 8 per group). The number and maximum diameter of the foci confirmed to be endometriotic by histopathology were recorded. To evaluate the accuracy of the probe, ten endometriotic model mice were intraperitoneally injected with GnRHa-ICG, followed by GFP and NIRF imaging. The GFP-positive tissues were resected under the guidance of NIRF imaging, and the sensitivity and specificity of the probe were calculated with the histopathology examination as a reference.

### 2.10. In Vivo Toxicity Tests

GnRHa-ICG toxicity was determined in BALB/c mice (*n* = 3). Two groups received intraperitoneal injections of 1.0 mg/kg GnRHa-ICG and were followed for 2 and 120 h. The control group received the vehicle alone. Blood was drawn to assess alanine transaminase (ALT), aspartate transaminase (AST), blood urea nitrogen (BUN), creatinine (CREA), white blood cells (WBC), and red blood cells (RBC). Heart, lung, liver, spleen, and kidney tissues were harvested for HE staining.

### 2.11. Statistical Analysis

Student's *t*-test was used to compare the intensities. Differences were considered significant at *P* < 0.05 and are reported as the mean ± SD. All statistical analyses were performed using GraphPad Prism 8.0 (GraphPad Software, Inc.).

## 3. Results

### 3.1. GnRHR Expression in Human Endometriotic Tissues and Cell Lines

**Figure 1:**
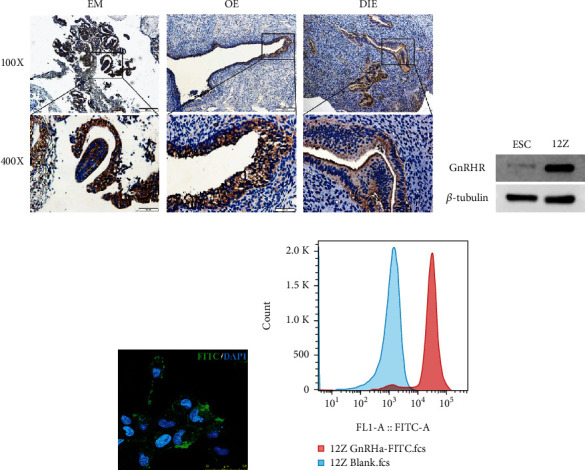
Expression of GnRHR in the endometriotic tissues and cell lines. (a) Representative IHC staining for GnRHR in human normal endometrium (EM), ovary endometriosis (OE), and deep infiltrating endometriosis (DIE). (b) Western blot showing GnRHR expression in immortalized human endometriotic epithelial cell line 12Z and endometrial stromal cells (ESC). (c) Representative fluorescence microscopy images and (d) flow cytometry analysis of 12Z cells after incubation with GnRHa-FITC. Scale bar, 50 *μ*m.

GnRHR expression was observed in 100% (6/6) of endometria (EM), 88.9% (40/45) of ovarian endometrioma (OE), and 88.4% (15/17) of deep endometriosis (DIE) samples by IHC analysis (Figure 1(a)). GnRHR was highly expressed in the human immortalized endometriotic epithelial cell line 12Z and negatively expressed in human endometrial stromal cell ESC, as determined by western blotting (Figure 1(b)).

### 3.2. The Binding Capacity of GnRHa Peptide to Endometriotic Cells

To evaluate whether GnRHa peptide binds to endometriotic cell lines, we labeled GnRHa peptide with FITC and incubated GnRHa-FITC with immortalized human endometriotic epithelial 12Z cells. Strong fluorescence signals of GnRHa-FITC were detected in 12Z cells via fluorescence microscopy and flow cytometry (Figures 1(c) and 1(d)).

### 3.3. Enhanced Cell Binding Capacity of GnRHa-ICG to Endometriotic Cells

**Figure 2:**
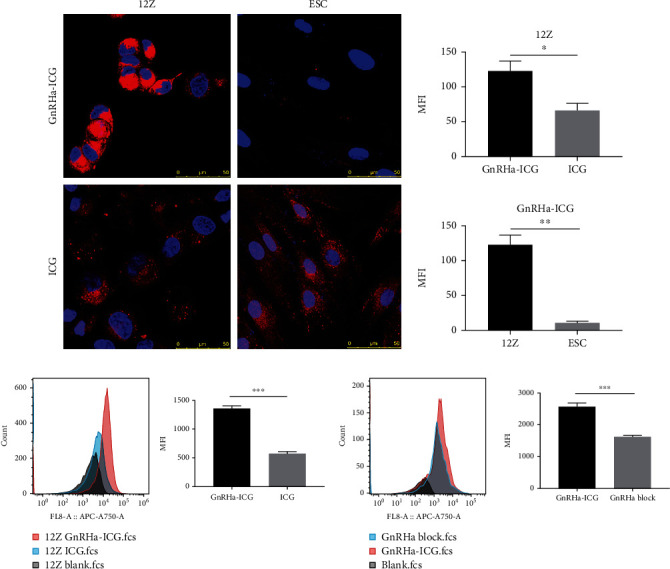
Cell binding capacity of GnRHa-ICG. (a) Comparison between GnRHa-ICG and ICG in 12Z and ESC cells. Scale bar, 50 *μ*m; magnification, ×1000. (b) Flow cytometry analysis of 12Z cells after incubation with GnRHa-ICG and ICG. (c) Flow cytometry analysis of 12Z cells after the GnRHa block (^∗^*P* < 0.01; ^∗∗^*P* < 0.001; ^∗∗∗^*P* < 0.0001).

To evaluate the binding capacity of GnRHa-ICG to endometriotic cells, we incubated GnRHa-ICG with the 12Z and ESC cell lines. A significant difference in fluorescence intensity was detected between GnRHR-positive 12Z cells and GnRHR-negative ESC (Figure 2(a)). The mean fluorescence intensity of GnRHa-ICG was 2.36-fold higher than that of ICG in the endometriotic 12Z cells (Figure 2(b)). After GnRHR blockade with unlabeled GnRHa peptides, GnRHa-ICG binding was significantly suppressed in GnRHR-positive 12Z cells (Figure 2(c)). Altogether, these results demonstrated the GnRHR-specific binding capacity of GnRHa-ICG.

### 3.4. Targeted Imaging of GnRHa-ICG in Mice Bearing Endometriosis

**Figure 3:**
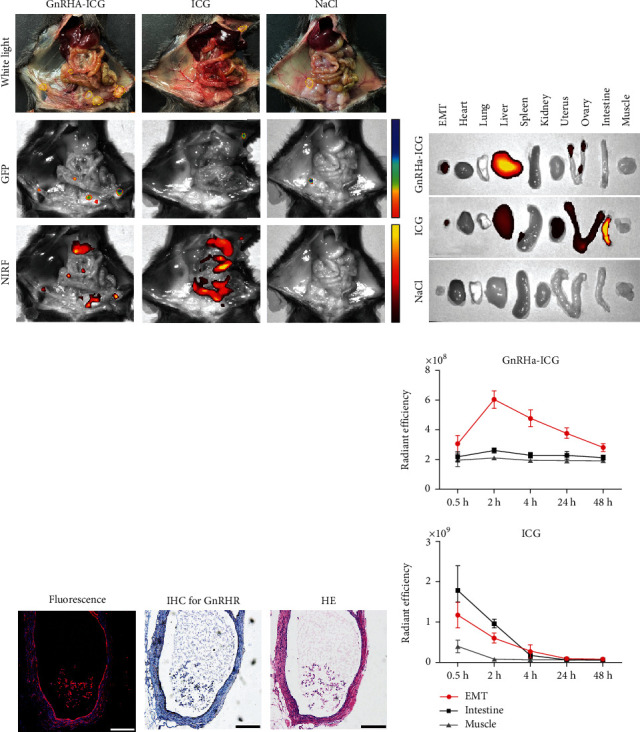
GnRHa-ICG targets endometriotic lesions. (a) Representative GFP and NIRF images of the pelvic and peritoneal cavity 2 h after injection of GnRHa-ICG, ICG, and NaCl in GFP mouse models for endometriosis. Yellow dotted lines indicate the EMT locations. (b) Ex vivo near-infrared fluorescence images of the EMTs and mouse organs 2 h after intraperitoneal injection of GnRHa-ICG, ICG, and NaCl. (c) Histopathological analysis of an EMT slice showing the colocalization of endometriotic cells, GnRHR expression, and GnRHa-ICG fluorescence. Scale bar, 250 *μ*m. (d) Comparison of fluorescence intensities between EMT, muscle, and intestine.

The fluorescence intensities of EMT and background tissues (muscle and intestine) per dose group are shown in Figure S1, indicating a low background in normal tissues. A dose of 1.0 mg/kg was used for the following experiments because of the higher SNR compared with 0.5 mg/kg and similar SNR with 1.5 mg/kg. Two hours postinjection with GnRHa-ICG or ICG and GFP and NIR fluorescence images of mouse abdomens were obtained and analyzed using the IVIS Lumina K imaging system (Figure 3(a)). More EMT foci were imaged under NIRF in the GnRHa-ICG group than in the white light group. The NIRF signals of the EMT foci were consistent with the GFP fluorescence signals from donor mice. Moreover, GnRHa-ICG specifically localized to EMT foci, whereas accumulation of ICG was mainly observed in the intestine.

Ex vivo NIRF images of the EMT foci and other organs demonstrated that the fluorescence signals were mainly concentrated in the EMT foci and liver after intraperitoneal injection of GnRHa-ICG, whereas they were predominantly concentrated in the intestine, liver, uterus, and kidney in the ICG group (Figure 3(b)). The fluorescence signal intensities of the ex vivo tissues and organs are shown in Figure S2. The EMT foci imaged by GnRHa-ICG were also confirmed by pathology and showed GnRHR expression (Figure 3(c)). Furthermore, GnRHa-ICG exhibited the highest fluorescence signals and SNR in the EMT foci at 2 h postinjection and a stable SNR between 0.5 and 48 h. However, the fluorescence signals of ICG were mainly concentrated in the intestine and decreased exponentially (Figure 3(d)). The poor SNR and rapid decay of ICG signals influenced the detection of the EMT foci.

### 3.5. Targeted NIRF Imaging-Guided Resection of Endometriotic Lesions

**Figure 4:**
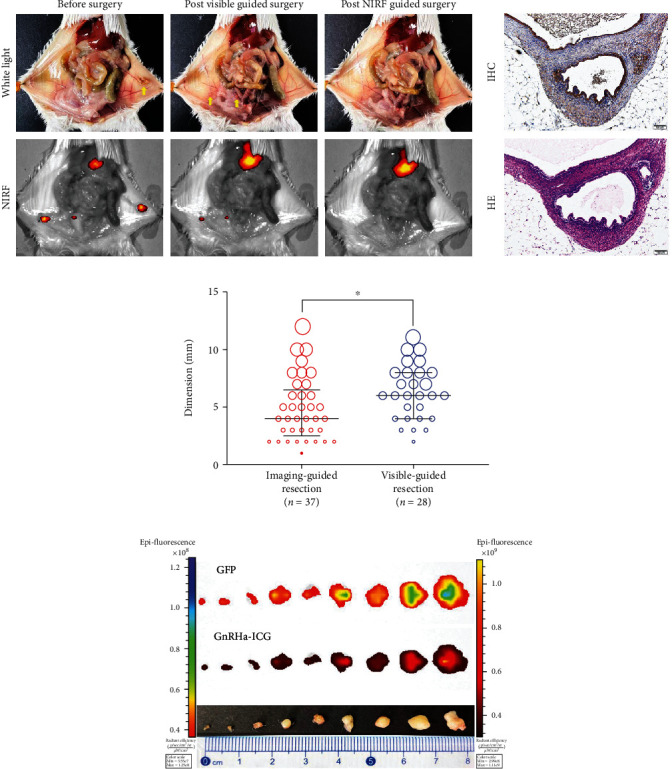
Targeted recognition capacity of GnRHa-ICG. (a) Comparison of recognition capacity to the EMTs between visible-guided surgery and NIRF-guided surgery. After visible surgical resection, no visible lesions were observed, but there were remaining hidden lesions removed under the guidance of NIRF. Yellow arrows indicate the EMT locations. (b) The tissues resected with NIRF-guided surgery were confirmed to be endometriotic tissues by histopathology. (c) Comparing the maximum diameters of the EMTs resected by imaging-guided surgery (red circles) and visible-guided surgery (blue circles) (37 vs 28 biopsies, *n* = 8 mice), and the median size is 3.8 ± 2.4 and 7.0 ± 2.9 mm, respectively (^∗^*P* < 0.01). (d) The recognition accuracy of GnRHa-ICG indicates that the probe can identify the lesions as small as 1 mm in diameter.

To further evaluate the clinical value of GnRHa-ICG, EMT resections were performed under the guidance of white light and NIRF 2 h after intraperitoneal injection of GnRHa-ICG (Figure 4(a)). Minute residual endometriotic lesions in the abdominal cavity after white light-guided surgery were found on NIRF imaging via GnRHa-ICG. These residual foci were resected under NIRF imaging and were pathologically confirmed to be endometriotic tissues (Figure 4(b)). In vivo and ex vivo imagings using a clinically applied intraoperative fluorescence navigation imaging system were the same as those of the IVIS Lumina K imaging system (Figure S3). The median diameter of the EMT lesions resected in the NIRF-guided group (3.8 ± 2.4 mm, *n* = 37) was significantly smaller than that in the white light-guided group (7.0 ± 2.9 mm, *n* = 28) (Figure 4(c)). Under the guidance of NIRF using GnRHa-ICG, the minimum detectable lesions were 1 mm in diameter (Figure 4(d)). GnRHa-ICG provided excellent sensitivity (97.3%) and acceptable specificity (77.8%), with pathological examination as a reference.

### 3.6. Biodistribution and Dynamics of GnRHa-ICG in Mice Bearing Endometriosis

**Figure 5:**
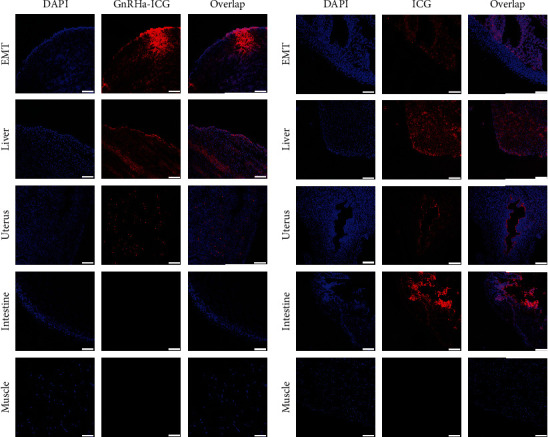
Comparison of biodistribution between GnRHa-ICG and ICG. Confocal microscopy of frozen sections of the EMTs and other organs in mice injected with (a) GnRHa-ICG and (b) ICG. Scale bar, 75 *μ*m.

To evaluate the biodistribution of the GnRHa-ICG probes, ex vivo EMTs and organs were collected 2 h postadministration. Confocal imaging confirmed the high accumulation of GnRHa-ICG in the EMT and liver, while ICG showed strong fluorescence signals in the intestine and liver and a weak signal in the EMT (Figure 5). These results are consistent with the in vivo fluorescence imaging.

**Figure 6:**
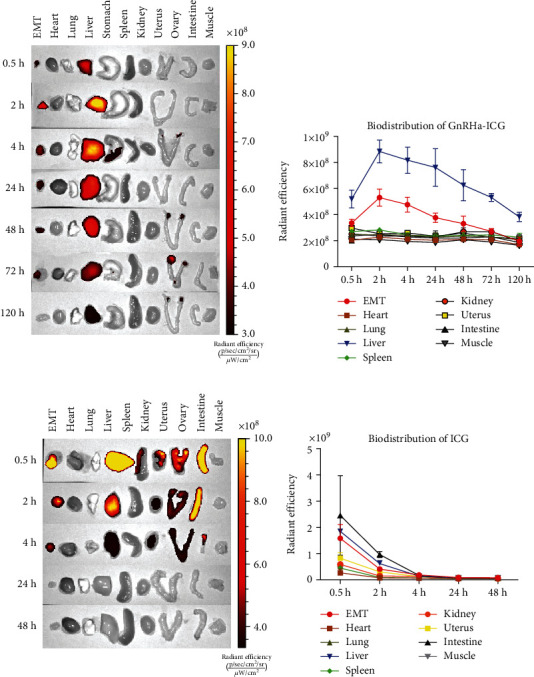
Dynamics and biodistribution of GnRHa-ICG and ICG. Representative fluorescence images of ex vivo endometriotic tissues and organs after injection of GnRHa-ICG (a) or ICG (c). Biodistribution of GnRHa-ICG (b) or ICG (d).

The dynamics of GnRHa-ICG and ICG in mice with endometriosis were evaluated. Mice were intraperitoneally injected with GnRHa-ICG or ICG and monitored for 48–120 h. As shown in Figures 6(a) and 6(b), the fluorescence signals of GnRHa-ICG reached the highest peak in EMTs and the liver at 2 h postadministration and then decreased gradually with a good SNR within 48 h. Liver clearance was the main metabolic pathway and was almost completely metabolized at 120 h postadministration. Although normal uterus and ovary tissues also showed a slight increase in fluorescence signals, indicating GnRHR-specific binding of the probe, the EMTs were high enough to differentiate them from those of normal reproductive tissues. Few fluorescent signals were detected in the intestines, kidneys, and other normal tissues. In contrast, the fluorescence signals of ICG were mainly concentrated in the intestine and liver and almost completely metabolized at 48 h postadministration (Figures 6(c) and 6(d)). In addition to the EMTs, the uterus and kidney also showed fluorescence signals owing to the nonspecificity of ICG.

### 3.7. Toxicity of GnRHa-ICG In Vitro and In Vivo

**Figure 7:**
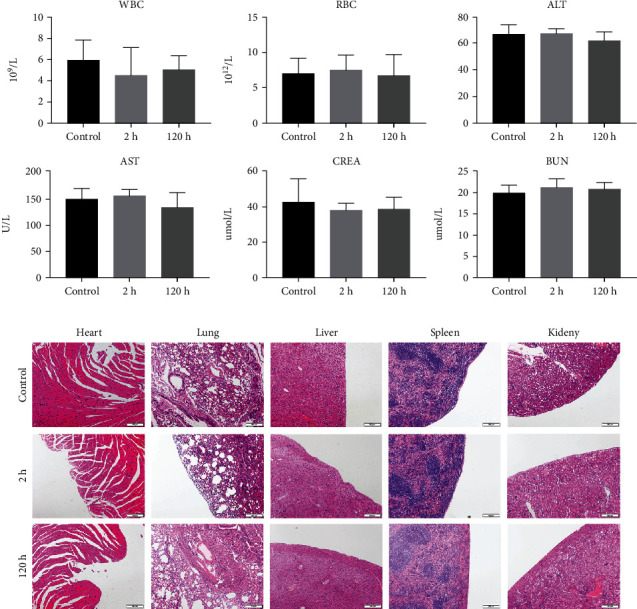
Toxicity of GnRHa-ICG in BALB/c mice. (a) Assessment of the liver panel, kidney panel, and blood cells. (b) HE staining of the heart, lung, liver, spleen, and kidney. Scale bar, 200 *μ*m.

To address concerns of possible side effects caused by GnRHa-ICG, we assessed the viability of GnRHR-positive 12Z cells after dose-gradient incubation with GnRHa-ICG. Exposure to a high concentration of GnRHa-ICG did not decrease the cell viability (Figure S4). In mice intraperitoneally injected with GnRHa-ICG or vehicle control, no significant differences were observed in WBC, RBC, ALT, AST, CREA, or BUN at 2 h and 120 h postinjection (Figure 7(a)). Histological examination of the main organs did not reveal any obvious damage (Figure 7(b)).

## 4. Discussion

In our study, we first reported intraoperative imaging and imaging-guided surgery using the targeted near-infrared fluorescent probe GnRHa-ICG in an endometriotic mouse model, which suggested a strong potential in clinical intraoperative imaging for endometriosis.

Over the past few decades, imaging approaches have been reported for the intraoperative detection of endometriosis; however, their diagnostic performance has been limited. Methylene blue, a widely used thiazide dye in surgical imaging, exhibits blue staining of peritoneal endometriotic lesions, but the colored peritoneal areas were not histopathologically confirmed [25, 26]. 5-Aminolevulinic acid (5-ALA), an endogenous nonproteinogenic amino acid, has been used to visualize bladder cancer [27], early-stage lung cancer [28], and malignant gliomas [29] using photodynamic detection. It has also been reported to help in the detection of red and white peritoneal endometriotic lesions, but not pigmented lesions [30], and its high rate of topical phototoxicity cannot be ignored [31].

Autofluorescence, the natural emission of light by biological structures, such as mitochondria and lysosomes [32], has been proven to be advantageous in the diagnosis of precancerous lesions in the bronchial tract [33] and bladder [34]. Buchweitz et al. reported the diagnostic value of autofluorescence imaging in the detection of red and occult peritoneal endometriotic lesions compared with white light, but no help in endometriomas and DIE [35]. Therefore, a specific imaging probe needs to be developed to improve diagnostic performance in endometriosis.

Targeted imaging of endometriosis can be performed by conjugating imaging agents with endometriotic-specific ligands (such as small molecules, peptides, proteins, and antibodies) [36, 37]. In 2006, 3-aminoethyl estradiol (EDL) was conjugated to glutamate peptide (GAP) to yield GAP-EDL (a functional estrogen ligand) and then labeled with ^99m^Tc to develop a radioactive imaging probe ^99m^Tc-GAP-EDL, which was first reported to target endometriotic lesions in a rabbit model of endometriosis [38]. Considering the superexpression of vascular endothelial growth factor (VEGF) in endometriosis [39–42], ^99m^Tc-labeled monoclonal anti-VEGF antibody (bevacizumab-^99m^Tc) was used as a radiopharmaceutical for endometriosis imaging in an endometriotic rat model in 2015 [43]. Moses et al. constructed silicon naphthalocyanine- (SiNc-) loaded polymeric nanoparticles (SiNc-NP) that efficiently delineated endometriotic lesions with NIR fluorescence signals in a mouse model of endometriosis in 2020 [44]. However, the reported targeted imaging probes have the universal shortcomings of radioactivity, insufficient targeting, or toxicity side effects, which restrict their clinical application.

Gonadotropin-releasing hormone receptor, which was mentioned in our study as GnRHR-I, is mainly expressed in the anterior pituitary and reproductive tissues, such as the breast, ovary, and prostate [45–47]. Higher GnRHR expression was observed in reproductive tissues than in other tissues from the GTEx portal database (data not shown). Moreover, high expression of GnRHR has been reported in endometriotic cells and tissues [18, 19], which is consistent with our results showing a nearly 90% expression rate in both ovarian endometrioma and deep infiltrating endometriosis. This finding suggests its potential as a novel imaging target in endometriosis.

NIRF imaging is an emerging real-time imaging technology that has been widely used in fluorescence-guided surgery because of its high penetration depth and low autofluorescence background [48]. ICG, the FDA-approved near-infrared fluorophore, has been widely used in clinical angiography and lymphography [49, 50]. ICG can visualize highly neovascularized endometriotic lesions under near-infrared fluorescence imaging, but its performance in the detection of endometriosis during surgery is inconsistent [51]. In our study, ICG showed high fluorescence in the liver and intestine and weak signals in the endometriotic lesions, indicating nonspecific imaging.

The use of NIRF imaging for molecularly guided surgical resection of cancers could reduce residual tumor burden and surgical morbidity associated with excising sufficient tissues to avoid positive surgical margins [52]. Based on our previous research [24], we conjugated GnRH peptides with a high affinity for binding to GnRHR with ICG to synthesize a targeted imaging probe, GnRHa-ICG. We demonstrated that GnRHa-ICG could specifically recognize endometriotic lesions from pelvic and peritoneal normal tissues and easily distinguish EMTs in mouse models of endometriosis. After intraperitoneal administration, GnRHa-ICG was mainly concentrated in the EMTs and liver, whereas ICG was mainly concentrated in the intestine and liver. We also observed that ICG exhibited a weak NIRF signal in the EMTs, but its fluorescence intensity decreased quickly within 24 h.

To further evaluate the target recognition capability of GnRHa-ICG for endometriosis, white light and NIRF-guided surgeries were performed. GnRHa-ICG could recognize tiny endometriotic lesions that could not be identified under white light, and the minimum detectable lesions were up to 1 mm in diameter. GnRHa-ICG has the potential to help localize tiny residual lesions that the naked eye cannot identify during surgery. Moreover, the signals of ICG decreased exponentially in mice after intraperitoneal administration, whereas GnRHa-ICG reached the highest concentration in the EMTs at 2 h and still had a good SNR within 48 h of administration. This period provides flexible administration time for clinical intraoperative imaging.

The toxicity of GnRHa-ICG was assessed, and no hematotoxicity and hepatotoxicity were observed in the study. The short-term administration of GnRHa-ICG is safe in vivo.

## 5. Conclusion

In summary, GnRHa-ICG specifically recognized and localized endometriotic lesions and showed high fluorescence signals from 2 to 48 h postadministration. Targeted NIRF imaging-guided surgery completely resected the endometriotic lesions with high accuracy. Future clinical applications might help surgeons accurately resect lesions to reduce the postoperative recurrence of endometriosis.

## Data Availability

All datasets generated for this study are included in the article/supplementary material.

## Supplementary Materials

Supplementary Materials Figure S1: dose-gradient analyses of GnRHa-ICG. (A) Mean fluorescence intensities of EMT and background tissues; (B) signal-to-noise ratio per dose group. Figure S2: fluorescence signal distribution of ex vivo tissues and organs after 2 h injection of GnRHa-ICG (A) and ICG (B). Figure S3: fluorescence imaging of endometriotic model mice with clinically applied intraoperative fluorescence navigation imaging system after 2 h injection of GnRHa-ICG (A) and ICG (B). The ex vivo tissues are EMT, heart, lung, liver, spleen, kidney, uterus and ovary, intestine, and muscle from left to right and top to bottom, respectively. Yellow dotted lines indicate the EMT locations. Figure S4: cell viability assay after dose-gradient incubation of GnRHa-ICG. Samples include no exposure to GnRHa-ICG (control) and a 48 h exposure to different concentrations of GnRHa-ICG.
