# Supplementary material for: Targeted Imaging of Endometriosis and Image-Guided Resection of Lesions Using Gonadotropin-Releasing Hormone Analogue-Modified Indocyanine Green
Source: Mol Imaging. 2023 Dec 4;2023:6674054. doi: 10.1155/2023/6674054 (PMC10713253; doi:10.1155/2023/6674054)
Supplement: Supplementary Materials — Figure S1: dose-gradient analyses of GnRHa-ICG. (A) Mean fluorescence intensities of EMT and background tissues; (B) signal-to-noise ratio per dose group. Figure S2: fluorescence signal distribution of ex vivo tissues and organs after 2 h injection of GnRHa-ICG (A) and ICG (B). Figure S3: fluorescence imaging of endometriotic model mice with clinically applied intraoperative fluorescence navigation imaging system after 2 h injection of GnRHa-ICG (A) and ICG (B). The ex vivo tissues are EMT, heart, lung, liver, spleen, kidney, uterus and ovary, intestine, and muscle from left to right and top to bottom, respectively. Yellow dotted lines indicate the EMT locations. Figure S4: cell viability assay after dose-gradient incubation of GnRHa-ICG. Samples include no exposure to GnRHa-ICG (control) and a 48 h exposure to different concentrations of GnRHa-ICG. [file 6674054.f1.docx]

**Supplementary Data**


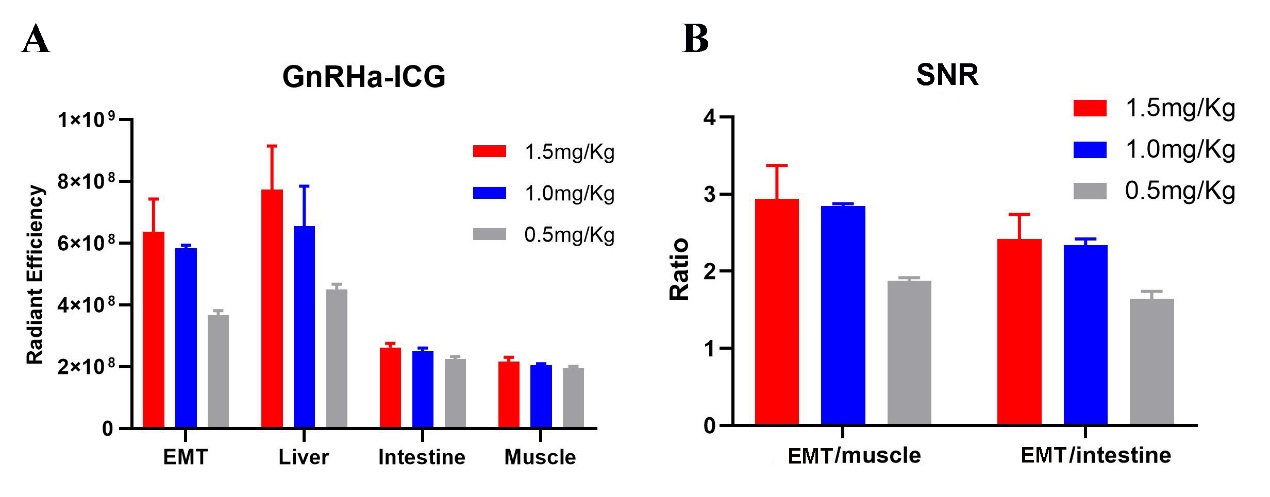


Figure S1 Dose gradient analyses of GnRHa-ICG. **(A)** Mean fluorescence intensities of EMT and background tissues; **(B)** Signal-to-noise ratio per dose group.


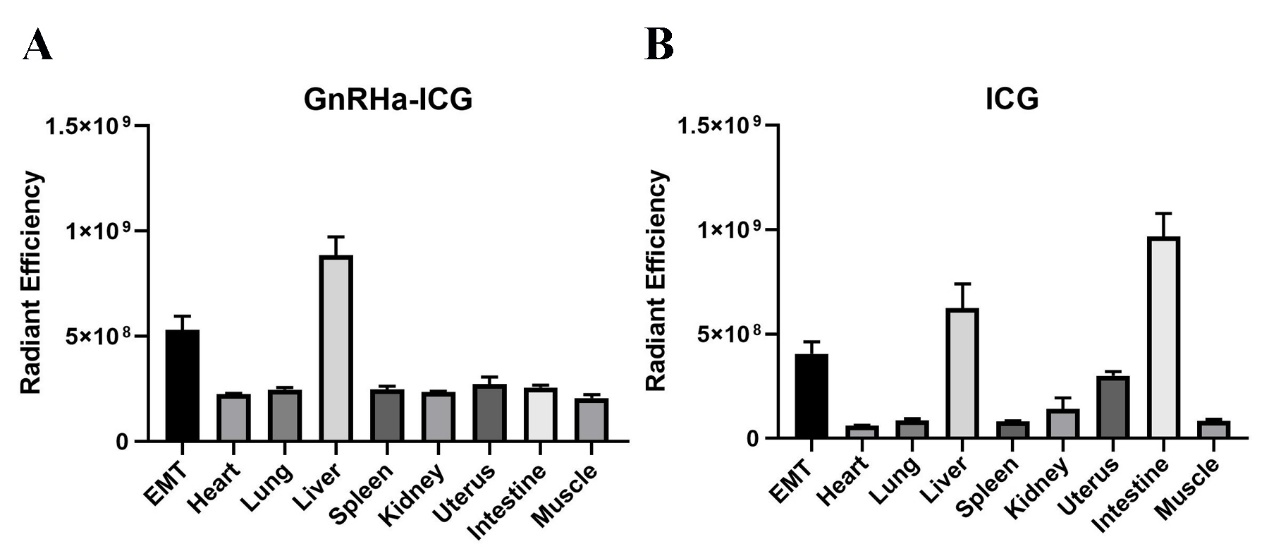


Figure S2 Fluorescence signal distribution of ex vivo tissues and organs after 2 h injection of GnRHa-ICG **(A)** and ICG **(B)**.


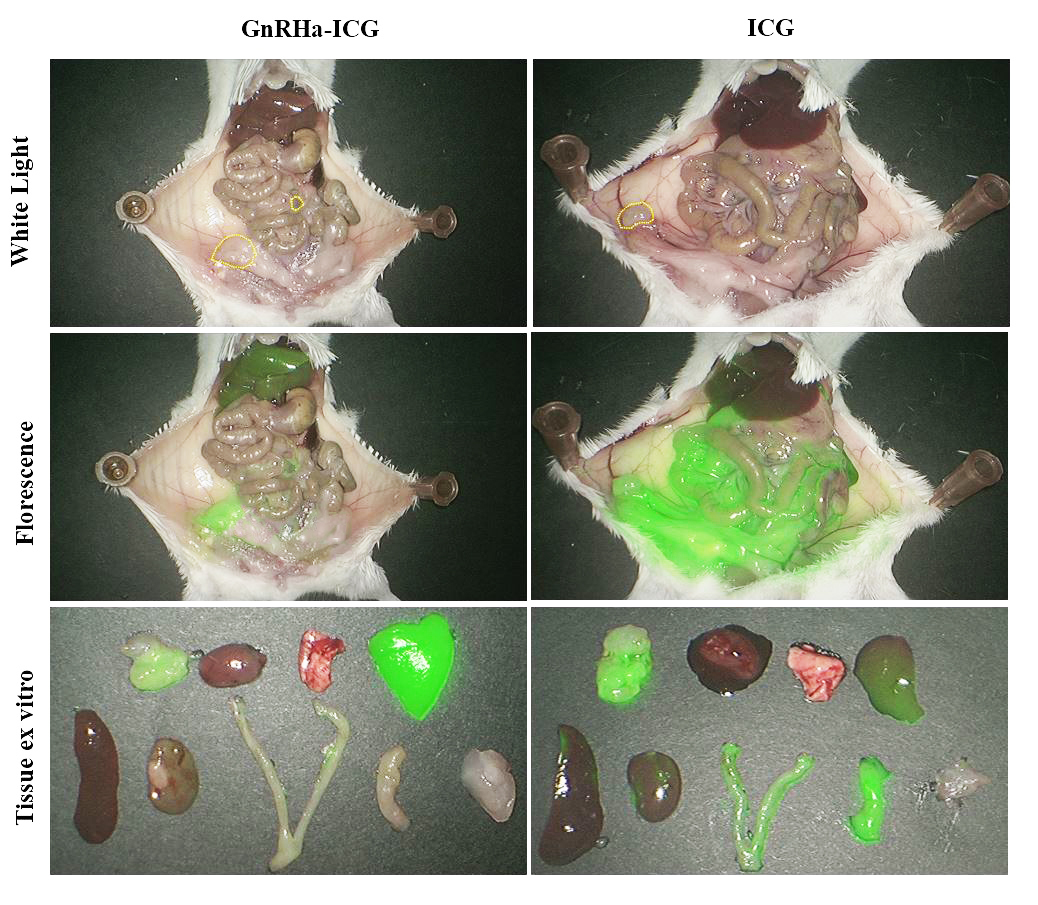


Figure S3 Fluorescence imaging of endometriotic model mice with clinically applied intraoperative fluorescence navigation imaging system after 2 h injection of GnRHa-ICG (**A**) and ICG (**B**).The ex vivo tissues are EMT, heart, lung, liver, spleen, kidney, uterus and ovary, intestine and muscle from left to right and top to bottom respectively. Yellow dotted lines indicate the EMT locations.

**
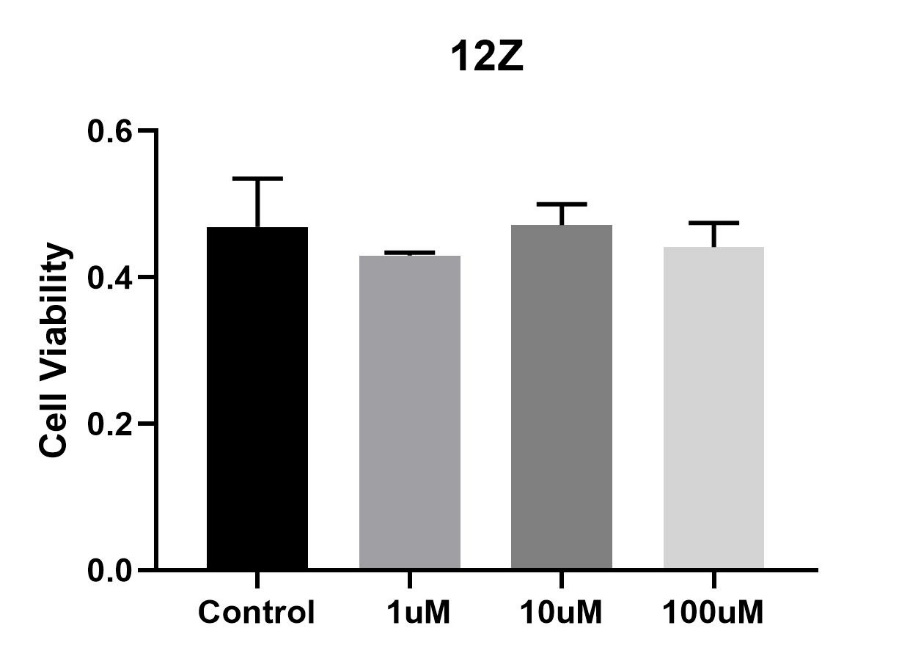
**

Figure S4 Cell viability assay after Dose gradient incubation of GnRHa-ICG. Samples include no exposure to GnRHa-ICG (control) and a 48-h exposure to different

concentrations of GnRHa-ICG.
